# Supplementary material for: Beyond the Microbiome: The Gut’s Role in Hypertension
Source: Function (Oxf). 2025 Sep 3;6(5):zqaf037. doi: 10.1093/function/zqaf037 (PMC12448473; doi:10.1093/function/zqaf037)
Supplement: zqaf037_review_manuscript_tracked_changes [file zqaf037_review_manuscript_tracked_changes.docx]

**Beyond the Microbiome: The Gut’s Role in Hypertension**

Wenjun Deng^1*^, Mengying Zhu^1,2*^, Isaac Lloyd^3^, Manaswini Nedunuri^3^, Chen Zhou^1^, Wenting Liu^1,2^, Yawen Li^1,2^, Qi Li^1,2^, Xinyue Wang^1,2^, Qiangxiang Zhang^4^, Tania Akter Jhuma^3^, Jing Li^1,5,#^, Tao Yang^3,#^

^1^ State Key Laboratory of Plant Diversity and Specialty Crops, Wuhan Botanical Garden, Chinese Academy of Sciences, Wuhan, 430074, China

^2^ University of Chinese Academy of Sciences, Beijing 100049, China.

^3^ Department of Physiology and Pharmacology, Center for Hypertension and Precision Medicine, College of Medicine and Life Sciences, University of Toledo, Toledo, Ohio, USA.

^4^ School of Medicine, Xiangyang Polytechnic, Xiangyang 441021, Hubei, China.

^5^ Hubei Jiangxia Laboratory, Wuhan 430299, Hubei, China.

*These authors equally contribute to this paper.

^#^ Correspondence to:

Jing Li, Ph.D.

E-mail: [lijing@wbgcas.cn](mailto:lijing@wbgcas.cn)

Tao Yang, Ph.D.

Email: [tao.yang2@utoledo.edu](mailto:tao.yang2@utoledo.edu)

**ABSTRACT**

This review emphasizes the importance of investigating the gut itself-beyond microbiota-centered studies in the context of hypertension. Since the initial discovery of the connection between gut microbiota and blood pressure regulation, research has increasingly focused on understanding the role of gut microbiota and exploring strategies to modify it for better blood pressure management. The intestine as an organ has received comparatively less attention. Yet, hypertension-associated intestinal pathological changes are well documented in both rodent models and human patients. Research to restore the intestinal function may serve as a valuable but unexplored therapeutic target. This underscores the need for a summary of our understanding of the gut’s intrinsic physiological and pathological roles in hypertension. To address this, we structured our review to (1) revisit the physiological functions of the intestine; (2) describe the pathological changes that are associated with hypertension; (3) summarize available current studies targeting to restore intestinal function for blood pressure control; (4) discuss knowledge gaps and future opportunities.

**KEYWORDS:** Hypertension; gut pathology; gut microbiota; gut-brain axis; 3D organoid

**Background briefing on hypertension and the gut microbiota**

Hypertension (HTN) is the leading preventable risk factor associated with an estimated 8.5 million deaths worldwide in 2015 due to cardiovascular disease, stroke, chronic kidney disease, and obstructive sleep apnea^1,2^. The prevalence of HTN increased significantly from 2000 to 2010, especially in low- and middle-income countries^3^. Despite the adoption of lifestyle modifications, the introduction of new therapies, and the implementation of comprehensive medical interventions, the prevalence of HTN-related comorbidities and mortality continues to rise.

The gastrointestinal (GI) tract is the habitat for trillions of microorganisms, named gut microbiota, which includes a diverse range of bacteria, archaea, fungi, and numerous viruses^4^. Homeostasis of gut microbiota is critical to maintaining the overall health of the host^6^. Disruption of this balance leads to significant alterations in gut microbial composition and function, leading to intestinal dysfunction, which has been extensively reviewed elsewhere^7-10^. The role of gut in HTN was initially introduced due to the discovery of linkage of the gut microbiota to HTN^11,12^. Studies linked gut dysbiosis-an imbalance in the gut microbiota-to HTN in rodent models and human patients^11-13^. Dietary interventions using probiotics, prebiotics, and synbiotics to restore gut homesostasis have been shown to lower blood pressure^18-23^.

Despite the great advances in understanding how gut microbiota impacts blood pressure, there remains a gap in comprehending critical pathological changes within the intestine associated with HTN. This review focuses on intestinal pathological changes associated with HTN, an important yet often overlooked perspective on cardiovascular health. Understanding the intestinal changes in response to HTN and associated imbalances in gut microbiota may provide novel strategies targeting the intestinal health for blood pressure management. Therefore, we aim to elucidate the key aspects of the GI involvement in hypertensive disease, while acknowledging that this review is not intended to be comprehensive on the gut microbiota.

**Physiological functions of the intestine**

To better discuss the changes and impacts in the etiology of HTN, we first recapitulate the physiological functions of the intestine, including the fundamental intestinal processing, its role as a physical and immunological barrier, and the interaction between the gut and brain. Then we will discuss the pathological changes in the intestine that are associated with HTN.

*Digestion, Absorption, and Elimination*

The gut is a major organ for nutrient digestion and absorption^24,25^. Approximately 60-70% of complex carbohydrates and 85% of fats are absorbed in the small intestine^26^. Bile aids in emulsifying fats, a process that is facilitated by peristalsis throughout the small intestine^27^. Emulsification is essential for preventing fat coagulation and forming an amphipathic mixture with bile salts, which enables digestive lipases to hydrolyze lipids, preparing them for absorption^27^. The digestion and absorption of lipids mainly conclude in the jejunum portion of the small intestine, where triglycerides are hydrolyzed by pancreatic lipase^28^. For carbohydrate digestion, sucrase-isomaltase and β-glycosidase exhibit high activity levels in the jejunum, while glucoamylase shows high activity in the ileum. Water absorption begins as chyme moves through the GI tract from the small intestine to the large intestine via peristalsis. This process, along with mineral absorption, solidifies the chyme into feces^26^. Salt, an important dietary factor in HTN, is absorbed mainly in the large intestine^29^.

*Physical barrier and immune regulation*

The intestinal layers are well organized for the protective functions of the gut. The outer mucosal layer, epithelial cells, and lamina propria separate the lumen from the connective tissue and contribute to the physical barrier, defending the gut against harmful substances and pathogens^30^. The gut epithelia constitute a single polarized cell layer, including a variety of specialized cells^31,32^. Goblet cells are specialized epithelial cells that secrete mucins to form the physical mucus layer^33^. Mucin 2, produced by goblet cells, is the major component of the mucus layer^34^. The mucus layer physically separates the gut microbiota from intestinal epithelial cells and immune cells to maintain gut homeostasis^34^. Meanwhile, goblet cells dynamically form specialized structures as goblet cell-associated passage (GAP), which facilitates the transfer of luminal antigens across the epithelium^35^. The underlying epithelial cells are interconnected via protein complexes, such as tight junction (TJ) proteins, which further seal the epithelial layer. Epithelial cells provide the highly selective nature of the intestines. Specific transport proteins and junctional complexes facilitate the entry of essential nutrients, water and salt while preventing harmful pathogens and toxins from entering^36^.

This highly dynamic nature of the physical barrier interacts closely with various aspects of the immune system. Dendritic cells and lymph tissue are located under the lamina propria. Luminal antigens from the GAP are processed by dendritic cells and presented to T-cells in lymph nodes, which then signal B cells to produce antibodies^37^. The dynamic process of antigen sampling in the epithelium is well controlled by neurotransmitter acetylcholine, which directs mucus secretion and GAP formation^36,37^. This allows goblet cells to sustain the protective mucus barrier as well as concurrently deliver the luminal information to lamina propria^38,39^. The lymphatic vessels in lamina propria are responsible for immune system surveillance, as part of a network that transports lymphocytes, antigens, and various pathogens within lymph nodes. This layer is critical for the immunological function in distinguishing between substances that should be tolerated or excluded, leading to intestinal homeostasis.

*Gut-brain axis*

The gut-brain axis plays a crucial role in blood pressure regulation, with the autonomic nervous system and immune system serving as key mediators of brain-gut communication^10,40^. The autonomic nervous system consists of the sympathetic and parasympathetic branches. The vagus nerve, a major component of the parasympathetic system, innervates several internal organs, including the heart, lungs, kidneys, liver, and GI tract^40,41^. Bi-directional signals travel along the vagus nerve, with afferent fibers conveying sensory information (such as satiety, nausea, and pain) and efferent fibers regulating motor functions (such as heart rate, GI contractions, and gastric acid secretion)^42^. Activation of parasympathetic pathways leads to the release of acetylcholine, which induces vasodilation and decreases heart rate, ultimately lowering blood pressure^43^. Immune cells, including macrophages, dendritic cells, T cells, B cells, and microglia, respond to acetylcholine through the α7 nicotinic acetylcholine receptor (α7nAChR)^44^(Figure 2). This interaction reduces the release of proinflammatory cytokines, contributing to the reduction of high blood pressure.

Gut microbial-derived short chain fatty acids (SCFAs) have been shown to modulate blood pressure *in vivo* through various mechanisms^45,46^. In the context of the gut-brain axis, studies show that propionate activates sympathetic outflows via G protein-coupled receptor 41^47^, while acetate and butyrate stimulate vagal afferent discharge^48,49^. However, the impact of such autonomic nervous system stimulation on blood pressure remains unclear, as blood pressure measurements are lacking in these studies. Peripheral information sensed by afferent pathways is relayed to the nucleus of the solitary tract (NTS), a key sensory nucleus that integrates this peripheral input^50^. Neuronal projections between the NTS and the paraventricular nucleus (PVN) of the hypothalamus, a central cardioregulatory region, play a role in blood pressure regulation^51,52^. Additionally, butyrate in cerebrospinal fluid can be detected by circumventricular organs, and intracerebroventricular injection of butyrate has been shown to lower blood pressure in rodents^53^. Indole, a gut microbial-derived metabolite of tryptophan, increases blood pressure through both peripheral and central mechanisms^54,55^. Also, indole activates vagal neuronal pathways through its effects on serotonin induction^56^.

**Pathological changes in the intestine in HTN**

Pathological changes in the intestine have been documented across multiple HTN models, highlighting the significance of the gut in cardiovascular health^57,58^. Intestinal wall fibrosis and increased muscle wall thickness are considered histopathological hallmarks in HTN^59^. In two important hypertensive models, spontaneously hypertensive rat (SHR)^57^ and chronic angiotensin (Ang) II-induced HTN^57^, studies have reported increased intestinal fibrosis, muscle wall thickness, and dysplasia of intestinal villi. Antibiotics (i.e., minocycline)^57^ and the first-line antihypertensive drugs (i.e., captopril^60^, candesartan^61^) are shown to decrease fibrosis and muscular layer thickness and increase villi length, which are associated with lower blood pressure. Figure 1 and Figure 2 summarize common pathological changes observed in HTN and their effects on downstream signaling pathways.

*Abnormal intestinal absorption and metabolism*

Gut microbial metabolites are critical molecules in the occurrence and development of HTN^62^. SCFAs^11,16^, bile acids^63,64^ and hydrogen sulfide (H_2_S)^65,66^ are generally considered beneficial for blood pressure homeostasis, while trimethylamine-N-oxide (TMAO)^67,68^ and lipopolysaccharide (LPS)^16,69^ have been identified as pro-hypertensive factors. Bile acids are synthesized in the liver, which can be conjugated and converted into secondary bile acids by the gut microbiota. Both primary and secondary bile acids can activate Takeda G protein-coupled receptor 5 (TGR5) and farnesoid X receptor (FXR) to regulate blood pressure^63,64^. Both receptors are present in the endothelial cells of blood vessels and activation of these receptors has been shown to promote vascular relaxation through the release of nitric oxide^70,71^. In addition, both receptors are mainly expressed in the liver, where they are heavily involved in energy expenditure, such as glucose and lipid metabolism^72^. Although metabolic diseases are interconnected to HTN, it remains unclear how TGR5 and FXR may contribute to HTN through its impact on energy expenditure.

H_2_S, produced by both colons and gut microbiota, also contributes to blood pressure control. Mice lacking cystathionine γ-lyase, an enzyme for H_2_S biosynthesis, develop HTN as early as 8 weeks of age^73^. Supplementation with H_2_S reduces blood pressure in these mice, SHR and L-NG-Nitro arginine methyl ester (L-NAME) induced hypertensive rats^74^. Mechanisms underlying its antihypertensive effects include activation of ATP-sensitive potassium channels (leading to vascular smooth muscle hyperpolarization), blockade of voltage-gated calcium channels (reducing calcium influx in vascular smooth muscle), and enhancement of nitric oxide-induced vasodilation^75^.

In contrast, TMAO, a gut microbiota-derived metabolite of choline, betaine, and L-carnitine, induces HTN by increasing oxidative stress and vascular inflammation, both of which lead to endothelial dysfunction^67,68^. Similarly, LPS, a component of gram-negative bacterial cell wall, activates Toll-like receptor 4 and triggers immune responses. Upon translocation into systemic circulation, LPS induces excessive activation of immune cells and release of inflammatory mediators, which ultimately elevates blood pressure^76^. In addition, high dietary salt intake is a well-established risk factor for HTN. The mechanisms by which salt contributes to HTN have been extensively reviewed elsewhere^77^.

Abnormal intestinal absorption of salt and gut microbiota-derived metabolites may contribute to HTN. For example, pharmacological inhibition of intestinal sodium/hydrogen exchanger 3 (NHE3) has been shown to increase fecal sodium excretion and water content^78^. This change was associated with a sustained reduction in systolic blood pressure and attenuation of cardiac hypertensive end-organ damage in hypertensive rats^78,79^, suggesting that intestinal absorption of salt is important in blood pressure regulation.

SCFAs have been found to accumulate in the gut in HTN^53,80,81^. The butyrate levels in stool samples were higher in the SHR than those of Wistar Kyoto (WKY) rats^53^. However, the circulatory level of butyrate was significantly lower in the SHR^53^. This finding was associated with a reduction in the expression of butyrate transporter Slc5a8 in the colon^53^. In humans, two independent studies reported higher butyrate levels in stool samples from hypertensive patients compared to normotensive controls^80,81^. However, the absorption of butyrate has not been studied in humans. Tryptophan catabolism occurs through three major pathways including the host-mediated serotonin and kynurenine pathway and the gut microbiota-mediated indole pathway, which collectively regulate blood pressure through distinct yet interconnected mechanisms^82,83^. In serotonin pathway, tryptophan hydroxylase-mediated conversion of tryptophan to serotonin exerts dual effects, including vasoconstrictor and vasodilator properties^84^. Serotonin can directly activate its selective receptors to induce vasoconstriction and vasodilation or bind to endothelial receptors, eliciting the production of nitric oxide resulting in vasodilation^83^. In kynurenine pathway, tryptophan catabolism converts tryptophan to L-kynurenine in immune cells and intestinal epithelial cells via indoleamine 2,3-dioxygenase-1 (IDO1) or tryptophan 2,3-dioxygenase (TDO), which is a potent vasodilator that induces hypotension^82^. For the gut microbiota-mediated indole pathway, tryptophan is metabolized to tryptamine and signaling-active indole and derivatives. Indole has been shown to be pro-hypertensive in rodents^54^. Also, colonic indole elevates portal blood pressure, thereby affecting intestinal inflammation and hemostasis through the regulation of the gut-vascular barrier in rats^54^. Trasient blood pressure responses to tryptophan injection varies significantly depending on host species, delivery route, dosages, etc^85^. This may be due to the complexity of tryptophan receptors and tryptophan metabolism pathways^85,86^. Dietary treatment of tryptophan lowers blood pressure in both SHR^87^ and Dahl salt sensitive hypertensive rats^88^. Salt reduces tryptophan absorption, resulting in a lower level of tryptophan in circulation and a higher level in feces, which results in excessive indole production by gut microbiota^55^. Therefore, absorption of nutrients (SCFAs, salt, tryptophan) is closely associated with blood pressure regulation.

*Decreased goblet cells*

In conventional animal models, the proportion of goblet cells relative to total epithelial cells gradually increases from approximately 4% in the duodenum to about 16% in the descending colon^31^. However, the cecum of germ-free mice developed fewer goblet cells than conventional mice, indicating that gut microbiota is critical for the differentiation and maturation of goblet cells^89^. Studies have demonstrated a decrease in the number of goblet cells as well as thinner mucus layer in many animal models of HTN^57,90^ . A reduction in the thickness of mucus layer increases the likelihood of bacterial penetration. Consequently, the deceased number and impaired function of goblet cells enhanced microbial-epithelial interactions, triggering excessive immune responses in the host^91^. These events may, in turn, lead to gut microbiota imbalance with a bloom of pathogenic bacterial groups, further goblet cell disruption and sustained immune activation^10^. Therefore, goblet cell dysfunction initiates a cascading effects that ultimately compromise gut barrier integrity and promote immune activation^92^. However, studies on goblet cells are heavily focused on their role as a mucus producer. The role of goblet cells in GAP has never been explored in the context of HTN.

*Increased intestinal permeability and immune activation*

Beneath the mucus layer, gut epithelium is a physical and functional barrier sustained by TJ proteins such as occludins, claudins, junctional adhesion molecules, and zonula occludin proteins^36^. Disruption of the barrier leads to increased intestinal permeability, commonly referred to as “leaky gut”^34,57^. Measurement of plasma levels of intestinal fatty acid-binding protein (I-FABP), a protein primarily synthesized in the gut epithelium, is widely used to assess intestinal integrity^93^. Similarly, plasma levels of LPS and zonulin are also biomarkers of leaky gut. In hypertensive patients, significant increases in plasma levels of I-FABP, LPS, and zonulin were found^94^. Also, the levels of zonulin and systolic blood pressure were positively correlated^94^, suggesting that permeable gut may lead to high blood pressure. Another study involving 106 hypertensive subjects also reported impaired gut epithelial barrier function characterized by elevated plasma levels of LPS and diamine oxidase, an enzyme primarily in the intestine for histamine catabolism^95^. In several experimental models of HTN (i.e., SHR^57^, obstructive sleep apnea -induced HTN^90^, chronic AngII-induced HTN^57^), impaired gut epithelial integrity and increased permeability were found. Specifically, serum fluorescein isothiocyanate (FITC)-dextran was measured after its oral gavage in rodents. A higher level of FITC-dextran indicates a more permeable gut of SHR and AngII-induced HTN^16,57,96^. In line with this, the expression of TJ proteins was lower in these hypertensive animal models^97^.

Disruption in the intestinal barrier results in an influx of antigens from the gut microbiota. Overactivation of immune cells at the interface and their trafficking to extra-intestinal sites result in end-organ inflammation and even damage^98^. Compared with WKY, SHR exerted higher intestinal inflammation, with increases in macrophage (CD68^+^), T lymphocytes (CD3^+^), and proinflammatory cytokines *Il1b* and *Tnf*^57^. Studies on the colonic organoids and isolated epithelium demonstrated that the expression of most genes involved in the antigen presentation pathway was significantly downregulated in the SHR compared to WKY, resulting in ineffective immune responses to gut dysbiosis and the altered luminal environment in HTN^97,99^. In another model, exposure to a high-salt diet after administering Ang II resulted in an accumulation of macrophages and lymphocytes in the kidney, subsequently elevating blood pressure levels^100^.

Dendritic cells play a crucial role in immune-dependent blood pressure elevation by interacting with T cells to generate memory effector T cells to promote HTN^101^. T helper 17 cells are also associated with HTN development in animal models through the production of interleukin-17 (IL-17)^102,103^. Furthermore, IL-17 knockout mice and IL-17 receptor blockade displayed the reduction of blood pressure, implicating an important role of IL-17 in regulating blood pressure^104^.

B cells received comparatively less attention in HTN research. SHRs have recently been characterized by an imbalanced immunoglobulin composition with increased levels of IgM and IgG alongside diminished levels of IgA^105^. IgA is abundantly produced in the GI tract, regulating the gut microbiota composition by neutralizing toxins and inducing immune exclusion^106^. We employed IgA-sequencing to identify a significant decrease in IgA-coated microbiota in SHRs^107^, suggesting that the deficiency in both the amount and function of IgA may contribute to gut dysbiosis and HTN development^105^. Furthermore, increased gut permeability facilitated the release of pro-inflammatory factors into the bloodstream in SHRs^57^, causing low-grade systemic inflammation and neuroinflammation^57,108^. While these inflammatory responses are necessary for tissue defense, their overactivation may lead to diseases such as HTN.

*Imbalance in autonomic system*

Enhanced sympathetic activity and decreased parasympathetic activity have been observed in the animal models of HTN through the autonomic influence on bone marrow activity, which are associated with gut pathophysiology, gut microbial dysbiosis, and overall metabolic changes^41^. Sympathetic nervous system regulates intestinal motility and local immune responses, with increased sympathetic excitability in the early stage of HTN^109^. Microglial activation in the PVN, an autonomous brain region, contributes to the sympathetic activation and blood pressure elevation in both SHR and Ang II infusion HTN^110,111^. Suppression of inflammation in the PVN using a tetracyclin-3, a derivative of tetracycline with potent anti-inflammatory activity, inhibited microglial activation, normalized sympathetic nerve activity, attenuated HTN and, importantly, alleviated intestinal histopathological alternations^108^. This supports the important role of gut-brain axis in HTN and suggests that mitigation of this dysfunctional axis by suppression of inflammation in the PVN can attenuate gut pathology and elevated blood pressure in the PVN.

**Restoration of intestinal barrier for blood pressure control**

As previously discussed, intestinal pathophysiological changes are commonly observed in HTN models^57^. These disruptions impair normal gut functions, such as nutrient absorption, immune regulation, and microbial balance, all of which contribute to elevated blood pressure. This raises the question of whether restoring intestinal function would help lower blood pressure in HTN. However, few studies have directly addressed this approach, revealing a significant gap in the research field.

Several first-line antihypertensive drugs have shown beneficial effects on the gut. These include angiotensin converting enzyme inhibitor, captopril, and Ang II type 1 receptor blocker, candesartan. Although not directly targeting the gut, significant improvement in the gut integrity and barrier function associated with lowered blood pressure was observed in the HTN rodents that were treated with these drugs^57,60,61^.

Approaches to target gut microbiota for HTN also demonstrate beneficial effects on the gut. For instance, oral administration of the probiotic *Bifidobacterium breve* CECT7263 attenuated HTN and improved intestinal barrier function in deoxycorticosterone acetate -salt rats^23^. Long-term administration of kefir, which contains several strains of probiotic bacteria, has been shown to lower blood pressure and attenuate gut pathology in SHRs^22^.

There are only a few studies designed to directly treat the gut for HTN. Mei et al. reported that a repurposed drug 5-aminosalicylic acid effectively lowered blood pressure and increased colonic energy metabolism in Dahl salt-sensitive rats^112^. 5-aminosalicylic acid is a peroxisome proliferator-activated receptor γ agonist approved by U.S. Food and Drug Administration for inflammatory bowel diseases. Importantly, it exerts effects on the colonic epithelium topically, rather than systemically^113^. This suggests that 5-aminosalicylic acid-induced energy metabolism is beneficial to blood pressure control in colon. Colonocyte of germfree mice is a great model of energy deprivation. Due to the lack of gut microbiota-derived SCFA butyrate, a primary energy source for colon, colonocytes from germ-free mice were in an energy-deprived state and exhibited decreased expression of enzymes that catalyze key steps in intermediary metabolism. Butyrate was able to rescue the deficits in mitochondrial respiration and prevent it from undergoing autophagy^114^. In HTN, SCFA butyrate improved gut pathology and barrier function, and suppressed AngII-induced HTN^115^. Using the colonic organoids derived from SHRs and subjects with high blood pressure^99,116^, butyrate was found to upregulate the expression of TJ proteins, suggesting a direct effect of butyrate on the gut epithelium.

Gut microbe engineering is a promising tool for the treatment of chronic diseases^117^. Genetically engineered *Lactobacillus* expressing angiotensin converting enzyme (ACE) 2 has been shown to lower blood pressure in female *Ace2* knockout rats, linked to a specific reduction in colonic AngII, but not renal AngII^55^. Indeed, this engineered ACE2 expressing *Lactobacillus* strain has been shown to restore the intestinal expression ACE2, resulting in a preservation of gut barrier integrity^118^. These studies prove the feasibility of targeting the intestine to mitigate high blood pressure.

**Knowledge gaps, challenges and opportunities**

*Knowledge gaps*

Altered gut epithelial permeability is linked to the development of HTN. Previous discussion has demonstrated that intestinal permeability is one of the pathological mechanisms for HTN. However, there are limited studies designed to restore gut function for blood pressure control. Abnormal absorption and impaired barrier integrity are two major mechanisms involved in blood pressure regulation. Abnormal intestinal absorption of key metabolites has been implicated in HTN, including reduced uptake of butyrate^53^ and tryptophan^55^, and increased intestinal salt absorption^78,79^. However, the absorption dynamics of other blood pressure-regulating metabolites—such as TMA/TMAO, H_2_S, and bile acids—remain largely unexplored. Given their primary role in modulating vascular function, understanding their absorption is critically important.

Impaired intestinal barrier permits the translocation of gut-derived antigens, leading to heightened immune activation that contributes to elevated blood pressure. Current evidence indicates that a diet rich in fiber, intake of butyrate and probiotics, improves gut barrier integrity and regulates blood pressure^119^. However, there are few studies on the use of intestinal stem cells to replenish epithelial cells and restore barrier function. Intestinal stem cells, located at the base of intestinal crypts, are pivotal for the continuous renewal of the gut lining^120^. Under suitable *in vitro* conditions, these stem cells can be cultured into three-dimensional (3D) structures known as intestinal organoids that exhibits self-renewal capacity and exhibit polarized epithelial cells, TJ, mucus secretion, and stable epithelial phenotype and genotype for physiological functions ^121^. For studies on inflammatory bowel disease (IBD), organoids obtained from patients outperformed the Caco-2 cell line and effectively modeled the intestinal epithelium of IBD patients^120^. However, there remains a knowledge gap regarding their potential to restore gut barrier function and reduce blood pressure.

The NIH policy to include sex as a biological variable in all NIH-funded studies was implemented in 2016. Sex differences in intestinal function in the development of HTN are rarely reported^55,122-126^. Salt modulates tryptophan metabolism in a sex-specific manner. In males, salt reduces its absorption and enhances microbial production of indole from tryptophan. In females, salt does not affect absorption, resulting in more conversion to kynurenine by the host^55^. By measuring plasma lactulose and L-rhamnose ratio, a biomarker for intestinal permeability, studies reported that women presented with higher intestinal permeability^127,128^. Notably, little is known about the sex differences in intestinal barrier and goblet cells in the development of HTN. Also, Sex specific roles of the immune system have been well summarized elsewhere^120^. However, the understanding of sex differences in the intestine and mucosal immunity concerning HTN remains poorly understood and warrants further study.

*Challenges and opportunities*

Mechanistic studies on intestinal diseases relied on cell lines and animals^120^. However, there are no proper cell lines with HTN characteristics. Gut tissues collected from rodent models of HTN have been used to study the pathological changes in HTN. Animal studies face challenges such as high costs, long experimental cycles and variable factors (i.e., innervation, gut microbiota)^129^. Additionally, the NIH announced to encourage various approaches, rather than exclusively animal models for experimental design. These challenge current studies in HTN research. Intestinal organoids^130^ may provide an alternative and an opportunity to study HTN-associated intestinal changes. The cultured 3D organoid from HTN models provide a physiologically relevant model to investigate these HTN-associated pathophysiological mechanisms^99,131^. Importantly, ^121120^the intestinal organoids reflect the genetic characteristics of the individual from which they originated, which allows for the investigation of key biological variables such as sex and age.

Current studies revealed that colonic organoids from SHRs exhibit distinct transcriptomic profiles compared to normotensive WKY rats^99^, with reduced expression of genes related to immune responses, including antigen presentation, epithelial renewal^99^. Butyrate supplementation restored these immunity-related gene expression in SHR organoids^99^. What’s more relevant is that butyrate can also repair the deficient immune responses in the colonic organoids from patients with high blood pressure^116^. Additionally, minocycline, an anti-inflammatory antibiotic, rescued impaired expression of immunity-related genes in colonic organoids from patients with high blood pressure^132^. These support the use of organoids as a valuable platform to study the mechanisms for HTN-associated gut dysfunction (Figure 3).

**Conclusions**

Pathophysiological changes have been reported in multiple rodent HTN models. However, studies focusing on restoring gut function for blood pressure control are lacking. This review summarizes gut physiopathology in HTN, interventions to restore intestinal function for blood pressure control, and key knowledge gaps in the field. We also discuss recent advances in HTN-related intestinal organoid models and emerging evidence on sex differences in intestine during HTN. Collectively, targeting the intestine for better management of blood pressure is a promising but underexplored research area. The utilization of organoid models, combined with hypertensive rodent models, offers a powerful approach to uncover the role of gut in HTN.

**Data availability statement**

Data sharing is not applicable to this article as no new data were created or analyzed in this study.

**Declaration**

ChatGPT was used to refine the language for fluency and grammar. The authors reviewed and edited the content to ensure accuracy and take full responsibility for the final version.

**Acknowledgments**

T.Y. is supported by NIA/NIH R21AG079357. J.L. is supported by the Research Grant from Wuhan Botanic Garden (E2559901), the Natural Science Foundation of Hubei Province (2024AFA099), and the Key R&D Program of Hubei Jiangxia Laboratory (JXBS005 and JXBS010). W.J.D is supported by the National Natural Science Foundation of China (NSFC Accession No.32401053).

**References**

1. Zhou B, Perel P, Mensah GA, Ezzati M. Global epidemiology, health burden and effective interventions for elevated blood pressure and hypertension. *NATURE REVIEWS CARDIOLOGY.* 2021;18 (11):785-802. doi: 10.1038/s41569-021-00559-8.

2. Mills KT, Stefanescu A, He J. The global epidemiology of hypertension. *Nat Rev Nephrol.* 2020;16 (4):223-237. doi: 10.1038/s41581-019-0244-2.

3. Mills KT, Bundy JD, Kelly TN, et al. Global Disparities of Hypertension Prevalence and Control: A Systematic Analysis of Population-Based Studies From 90 Countries. *Circulation.* 2016;134 (6):441-450. doi: 10.1161/CIRCULATIONAHA.115.018912.

4. O'Riordan KJ, Collins MK, Moloney GM, et al. Short chain fatty acids: Microbial metabolites for gut-brain axis signalling. *Molecular and Cellular Endocrinology.* 2022;546. doi: 10.1016/j.mce.2022.111572.

5. Qin J, Li R, Raes J, et al. A human gut microbial gene catalogue established by metagenomic sequencing. *Nature.* 2010;464 (7285):59-65. doi: 10.1038/nature08821.

6. Wu G, Xu T, Zhao N, et al. A core microbiome signature as an indicator of health. *Cell.* 2024;187 (23):6550-6565 e6511. doi: 10.1016/j.cell.2024.09.019.

7. Gao K, Wang PX, Mei X, Yang T, Yu K. Untapped potential of gut microbiome for hypertension management. *Gut Microbes.* 2024;16 (1):2356278. doi: 10.1080/19490976.2024.2356278.

8. Avery EG, Bartolomaeus H, Maifeld A, et al. The Gut Microbiome in Hypertension: Recent Advances and Future Perspectives. *Circ Res.* 2021;128 (7):934-950. doi: 10.1161/CIRCRESAHA.121.318065.

9. O'Donnell JA, Zheng TH, Meric G, Marques FZ. The gut microbiome and hypertension. *NATURE REVIEWS NEPHROLOGY.* 2023;19 (3):153-167. doi: 10.1038/s41581-022-00654-0.

10. Yang T, Richards EM, Pepine CJ, Raizada MK. The gut microbiota and the brain-gut-kidney axis in hypertension and chronic kidney disease. *Nat Rev Nephrol.* 2018;14 (7):442-456. doi: 10.1038/s41581-018-0018-2.

11. Yang T, Santisteban MM, Rodriguez V, et al. Gut dysbiosis is linked to hypertension. *Hypertension.* 2015;65 (6):1331-1340. doi: 10.1161/HYPERTENSIONAHA.115.05315.

12. Mell B, Jala VR, Mathew AV, et al. Evidence for a link between gut microbiota and hypertension in the Dahl rat. *Physiol Genomics.* 2015;47 (6):187-197. doi: 10.1152/physiolgenomics.00136.2014.

13. Li J, Zhao F, Wang Y, et al. Gut microbiota dysbiosis contributes to the development of hypertension. *Microbiome.* 2017;5 (1):14. doi: 10.1186/s40168-016-0222-x.

14. Marques FZ, Nelson E, Chu P-Y, et al. High-Fiber Diet and Acetate Supplementation Change the Gut Microbiota and Prevent the Development of Hypertension and Heart Failure in Hypertensive Mice. *Circulation.* 2017;135 (10):964-977. doi: 10.1161/circulationaha.116.024545.

15. Bartolomaeus H, Balogh A, Yakoub M, et al. Short-Chain Fatty Acid Propionate Protects From Hypertensive Cardiovascular Damage. *Circulation.* 2019;139 (11):1407-1421. doi: 10.1161/CIRCULATIONAHA.118.036652.

16. Kim S, Goel R, Kumar A, et al. Imbalance of gut microbiome and intestinal epithelial barrier dysfunction in patients with high blood pressure. *Clin Sci (Lond).* 2018;132 (6):701-718. doi: 10.1042/CS20180087.

17. R RM, Zheng T, Dinakis E, et al. Gut Microbiota Metabolites Sensed by Host GPR41/43 Protect Against Hypertension. *Circ Res.* 2025;136 (4):e20-e33. doi: 10.1161/CIRCRESAHA.124.325770.

18. Miura K, Greenland P, Stamler J, Liu K, Daviglus ML, Nakagawa H. Relation of vegetable, fruit, and meat intake to 7-year blood pressure change in middle-aged men: the Chicago Western Electric Study. *Am J Epidemiol.* 2004;159 (6):572-580. doi: 10.1093/aje/kwh085.

19. Jama HA, Rhys-Jones D, Nakai M, et al. Prebiotic intervention with HAMSAB in untreated essential hypertensive patients assessed in a phase II randomized trial. *NATURE CARDIOVASCULAR RESEARCH.* 2023;2 (1):35-+. doi: 10.1038/s44161-022-00197-4.

20. Lawrence J. Appel, Thomas J. Moore, Eva Obarzanek, et al. A Clinical Trial of The Effects of Dietary Patterns on BloodD Pressure. *The New England Journal of Medicine.* 1997;336 (16):1117-1124.

21. Zarezadeh M, Musazadeh V, Ghalichi F, et al. Effects of probiotics supplementation on blood pressure: An umbrella meta-analysis of randomized controlled trials. *Nutr Metab Cardiovasc Dis.* 2023;33 (2):275-286. doi: 10.1016/j.numecd.2022.09.005.

22. de Almeida Silva M, Mowry FE, Peaden SC, Andrade TU, Biancardi VC. Kefir ameliorates hypertension via gut-brain mechanisms in spontaneously hypertensive rats. *J Nutr Biochem.* 2020;77:108318. doi: 10.1016/j.jnutbio.2019.108318.

23. Robles-Vera I, Toral M, de la Visitación N, et al. Probiotics Prevent Dysbiosis and the Rise in Blood Pressure in Genetic Hypertension: Role of Short-Chain Fatty Acids. *Mol Nutr Food Res.* 2020;64 (6):e1900616. doi: 10.1002/mnfr.201900616.

24. Choct M. Managing gut health through nutrition. *Br Poult Sci.* 2009;50 (1):9-15. doi: 10.1080/00071660802538632.

25. McBurney MI. The gut: central organ in nutrient requirements and metabolism. *Canadian journal of physiology and pharmacology.* 1994;72 (3):260-265. doi: 10.1139/y94-040.

26. Delbaere K, Roegiers I, Bron A, et al. The small intestine: dining table of host-microbiota meetings. *FEMS Microbiol Rev.* 2023;47 (3). doi: 10.1093/femsre/fuad022.

27. Schonewille M, de Boer JF, Groen AK. Bile salts in control of lipid metabolism. *Curr Opin Lipidol.* 2016;27 (3):295-301. doi: 10.1097/MOL.0000000000000303.

28. Xu E, Chen C, Fu J, et al. Dietary fatty acids in gut health: Absorption, metabolism and function. *Anim Nutr.* 2021;7 (4):1337-1344. doi: 10.1016/j.aninu.2021.09.010.

29. Bailey MA, Dhaun N. Salt Sensitivity: Causes, Consequences, and Recent Advances. *Hypertension.* 2024;81 (3):476-489. doi: 10.1161/HYPERTENSIONAHA.123.17959.

30. Vancamelbeke M, Vermeire S. The intestinal barrier: a fundamental role in health and disease. *Expert Rev Gastroenterol Hepatol.* 2017;11 (9):821-834. doi: 10.1080/17474124.2017.1343143.

31. van der Flier LG, Clevers H. Stem cells, self-renewal, and differentiation in the intestinal epithelium. *Annu Rev Physiol.* 2009;71:241-260. doi: 10.1146/annurev.physiol.010908.163145.

32. Gerbe F, Jay P. Intestinal tuft cells: epithelial sentinels linking luminal cues to the immune system. *Mucosal Immunol.* 2016;9 (6):1353-1359. doi: 10.1038/mi.2016.68.

33. Yang S, Yu M. Role of Goblet Cells in Intestinal Barrier and Mucosal Immunity. *Journal of Inflammation Research.* 2021;Volume 14:3171-3183. doi: 10.2147/jir.S318327.

34. Snelson M, Vanuytsel T, Marques FZ. Breaking the Barrier: The Role of Gut Epithelial Permeability in the Pathogenesis of Hypertension. *Curr Hypertens Rep.* 2024;26 (9):369-380. doi: 10.1007/s11906-024-01307-2.

35. McDole JR, Wheeler LW, McDonald KG, et al. Goblet cells deliver luminal antigen to CD103+ dendritic cells in the small intestine. *Nature.* 2012;483 (7389):345-349. doi: 10.1038/nature10863.

36. Zihni C, Mills C, Matter K, Balda MS. Tight junctions: from simple barriers to multifunctional molecular gates. *Nat Rev Mol Cell Biol.* 2016;17 (9):564-580. doi: 10.1038/nrm.2016.80.

37. Ali A, Tan H, Kaiko GE. Role of the Intestinal Epithelium and Its Interaction With the Microbiota in Food Allergy. *Front Immunol.* 2020;11:604054. doi: 10.3389/fimmu.2020.604054.

38. Knoop KA, McDonald KG, McCrate S, McDole JR, Newberry RD. Microbial sensing by goblet cells controls immune surveillance of luminal antigens in the colon. *Mucosal Immunol.* 2015;8 (1):198-210. doi: 10.1038/mi.2014.58.

39. Gustafsson JK, Davis JE, Rappai T, et al. Intestinal goblet cells sample and deliver lumenal antigens by regulated endocytic uptake and transcytosis. *Elife.* 2021;10. doi: 10.7554/eLife.67292.

40. Yang T, Zubcevic J. Gut-Brain Axis in Regulation of Blood Pressure. *Front Physiol.* 2017;8:845. doi: 10.3389/fphys.2017.00845.

41. Al-Shalan HAM, Hu DL, Nicholls PK, Greene WK, Ma B. Innervation and nerve-immune cell contacts in mouse Peyer's patches. *Histol Histopathol.* 2020;35 (4):371-383. doi: 10.14670/HH-18-158.

42. Breit S, Kupferberg A, Rogler G, Hasler G. Vagus Nerve as Modulator of the Brain-Gut Axis in Psychiatric and Inflammatory Disorders. *Front Psychiatry.* 2018;9:44. doi: 10.3389/fpsyt.2018.00044.

43. Gordan R, Gwathmey JK, Xie LH. Autonomic and endocrine control of cardiovascular function. *World J Cardiol.* 2015;7 (4):204-214. doi: 10.4330/wjc.v7.i4.204.

44. Fujii T, Mashimo M, Moriwaki Y, et al. Expression and Function of the Cholinergic System in Immune Cells. *Front Immunol.* 2017;8:1085. doi: 10.3389/fimmu.2017.01085.

45. Pluznick JL, Protzko RJ, Gevorgyan H, et al. Olfactory receptor responding to gut microbiota-derived signals plays a role in renin secretion and blood pressure regulation. *Proc Natl Acad Sci U S A.* 2013;110 (11):4410-4415. doi: 10.1073/pnas.1215927110.

46. Welathanthree M, Keating DJ, Macefield VG, Carnevale D, Marques FZ, R RM. Cross-talk between microbiota-gut-brain axis and blood pressure regulation. *Clin Sci (Lond).* 2025;139 (9). doi: 10.1042/CS20240787.

47. Kimura I, Inoue D, Maeda T, et al. Short-chain fatty acids and ketones directly regulate sympathetic nervous system via G protein-coupled receptor 41 (GPR41). *Proc Natl Acad Sci U S A.* 2011;108 (19):8030-8035. doi: 10.1073/pnas.1016088108.

48. Simon Lal, Anthony J. Kirkup, Brunsden AM, Thompson DG, Grundy D. Vagal afferent responses to fatty acids of different chain length in the rat. *Am J Physiol Gastrointest Liver Physiol.* 2001;281:907–915.

49. Perry RJ, Peng L, Barry NA, et al. Acetate mediates a microbiome-brain-beta-cell axis to promote metabolic syndrome. *Nature.* 2016;534 (7606):213-217. doi: 10.1038/nature18309.

50. Bassi JK, Connelly AA, Butler AG, et al. Analysis of the distribution of vagal afferent projections from different peripheral organs to the nucleus of the solitary tract in rats. *J Comp Neurol.* 2022;530 (17):3072-3103. doi: 10.1002/cne.25398.

51. Affleck VS, Coote JH, Pyner S. The projection and synaptic organisation of NTS afferent connections with presympathetic neurons, GABA and nNOS neurons in the paraventricular nucleus of the hypothalamus. *Neuroscience.* 2012;219 (1-2):48-61. doi: 10.1016/j.neuroscience.2012.05.070.

52. Savic B, Murphy D, Japundzic-Zigon N. The Paraventricular Nucleus of the Hypothalamus in Control of Blood Pressure and Blood Pressure Variability. *Front Physiol.* 2022;13:858941. doi: 10.3389/fphys.2022.858941.

53. Yang T, Magee KL, Colon-Perez LM, et al. Impaired butyrate absorption in the proximal colon, low serum butyrate and diminished central effects of butyrate on blood pressure in spontaneously hypertensive rats. *Acta Physiol (Oxf).* 2019;226 (2):e13256. doi: 10.1111/apha.13256.

54. Huc T, Nowinski A, Drapala A, Konopelski P, Ufnal M. Indole and indoxyl sulfate, gut bacteria metabolites of tryptophan, change arterial blood pressure via peripheral and central mechanisms in rats. *PHARMACOLOGICAL RESEARCH.* 2018;130:172-179. doi: 10.1016/j.phrs.2017.12.025.

55. Bardhan P, Mei X, Lai NK, et al. Salt Responsive Gut Microbiota Induces Sex Specific Blood Pressure Changes. *Circ Res.* 2024. doi: 10.1161/CIRCRESAHA.124.325056.

56. Ye L, Bae M, Cassilly CD, et al. Enteroendocrine cells sense bacterial tryptophan catabolites to activate enteric and vagal neuronal pathways. *Cell Host Microbe.* 2021;29 (2):179-196 e179. doi: 10.1016/j.chom.2020.11.011.

57. Santisteban MM, Qi Y, Zubcevic J, et al. Hypertension-Linked Pathophysiological Alterations in the Gut. *Circ Res.* 2017;120 (2):312-323. doi: 10.1161/CIRCRESAHA.116.309006.

58. Zubcevic J, Richards EM, Yang T, et al. Impaired Autonomic Nervous System-Microbiome Circuit in Hypertension. *Circ Res.* 2019;125 (1):104-116. doi: 10.1161/CIRCRESAHA.119.313965.

59. Zhu Z, Xiong S, Liu D. The Gastrointestinal Tract: an Initial Organ of Metabolic Hypertension? *Cell Physiol Biochem.* 2016;38 (5):1681-1694. doi: 10.1159/000443107.

60. Yang T, Aquino V, Lobaton GO, et al. Sustained Captopril-Induced Reduction in Blood Pressure Is Associated With Alterations in Gut-Brain Axis in the Spontaneously Hypertensive Rat. *J Am Heart Assoc.* 2019;8 (4):e010721. doi: 10.1161/JAHA.118.010721.

61. Wu D, Tang X, Ding L, et al. Candesartan attenuates hypertension-associated pathophysiological alterations in the gut. *Biomed Pharmacother.* 2019;116:109040. doi: 10.1016/j.biopha.2019.109040.

62. Yang Z, Wang Q, Liu Y, et al. Gut microbiota and hypertension: association, mechanisms and treatment. *Clin Exp Hypertens.* 2023;45 (1):2195135. doi: 10.1080/10641963.2023.2195135.

63. Li C, Li J, Weng X, Lan X, Chi X. Farnesoid X receptor agonist CDCA reduces blood pressure and regulates vascular tone in spontaneously hypertensive rats. *J Am Soc Hypertens.* 2015;9 (7):507-516 e507. doi: 10.1016/j.jash.2015.04.006.

64. Shi H, Zhang B, Abo-Hamzy T, et al. Restructuring the Gut Microbiota by Intermittent Fasting Lowers Blood Pressure. *Circ Res.* 2021;128 (9):1240-1254. doi: 10.1161/CIRCRESAHA.120.318155.

65. Weber GJ, Pushpakumar S, Tyagi SC, Sen U. Homocysteine and hydrogen sulfide in epigenetic, metabolic and microbiota related renovascular hypertension. *Pharmacol Res.* 2016;113 (Pt A):300-312. doi: 10.1016/j.phrs.2016.09.002.

66. Tomasova L, Dobrowolski L, Jurkowska H, et al. Intracolonic hydrogen sulfide lowers blood pressure in rats. *Nitric Oxide.* 2016;60:50-58. doi: 10.1016/j.niox.2016.09.007.

67. Jiang S, Shui Y, Cui Y, et al. Gut microbiota dependent trimethylamine N-oxide aggravates angiotensin II-induced hypertension. *Redox Biol.* 2021;46:102115. doi: 10.1016/j.redox.2021.102115.

68. Liu M, Han QH, Yang JL. Trimethylamine-N-oxide (TMAO) increased aquaporin-2 expression in spontaneously hypertensive rats. *Clin Exp Hypertens.* 2019;41 (4):312-322. doi: 10.1080/10641963.2018.1481420.

69. Battson ML, Lee DM, Weir TL, Gentile CL. The gut microbiota as a novel regulator of cardiovascular function and disease. *J Nutr Biochem.* 2018;56:1-15. doi: 10.1016/j.jnutbio.2017.12.010.

70. Kida T, Tsubosaka Y, Hori M, Ozaki H, Murata T. Bile acid receptor TGR5 agonism induces NO production and reduces monocyte adhesion in vascular endothelial cells. *Arterioscler Thromb Vasc Biol.* 2013;33 (7):1663-1669. doi: 10.1161/ATVBAHA.113.301565.

71. Zhang R, Ran HH, Zhang YX, et al. Farnesoid X receptor regulates vascular reactivity through nitric oxide mechanism. *J Physiol Pharmacol.* 2012;63 (4):367-372. <https://www.ncbi.nlm.nih.gov/pubmed/23070085>.

72. Chiang JYL, Ferrell JM. Bile acid receptors FXR and TGR5 signaling in fatty liver diseases and therapy. *Am J Physiol Gastrointest Liver Physiol.* 2020;318 (3):G554-G573. doi: 10.1152/ajpgi.00223.2019.

73. Yang G, Wu L, Jiang B, et al. H2S as a physiologic vasorelaxant: hypertension in mice with deletion of cystathionine gamma-lyase. *Science.* 2008;322 (5901):587-590. doi: 10.1126/science.1162667.

74. Wang R. Roles of Hydrogen Sulfide in Hypertension Development and Its Complications: What, So What, Now What. *Hypertension.* 2023;80 (5):936-944. doi: 10.1161/HYPERTENSIONAHA.122.19456.

75. Munteanu C, Popescu C, Vladulescu-Trandafir AI, Onose G. Signaling Paradigms of H(2)S-Induced Vasodilation: A Comprehensive Review. *Antioxidants (Basel).* 2024;13 (10). doi: 10.3390/antiox13101158.

76. Grylls A, Seidler K, Neil J. Link between microbiota and hypertension: Focus on LPS/TLR4 pathway in endothelial dysfunction and vascular inflammation, and therapeutic implication of probiotics. *Biomed Pharmacother.* 2021;137:111334. doi: 10.1016/j.biopha.2021.111334.

77. Masenga SK, Wandira N, Cattivelli-Murdoch G, et al. Salt sensitivity of blood pressure: mechanisms and sex-specific differences. *NATURE REVIEWS CARDIOLOGY.* 2025. doi: 10.1038/s41569-025-01135-0.

78. Linz B, Hohl M, Reil JC, Böhm M, Linz D. Inhibition of NHE3-mediated Sodium Absorption in the Gut Reduced Cardiac End-organ Damage Without Deteriorating Renal Function in Obese Spontaneously Hypertensive Rats. *JOURNAL OF CARDIOVASCULAR PHARMACOLOGY.* 2016;67 (3):225-231. doi: 10.1097/FJC.0000000000000336.

79. Linz D, Wirth K, Linz W, et al. Antihypertensive and laxative effects by pharmacological inhibition of sodium-proton-exchanger subtype 3-mediated sodium absorption in the gut. *Hypertension.* 2012;60 (6):1560-1567. doi: 10.1161/HYPERTENSIONAHA.112.201590.

80. Huart J, Leenders J, Taminiau B, et al. Gut Microbiota and Fecal Levels of Short-Chain Fatty Acids Differ Upon 24-Hour Blood Pressure Levels in Men. *Hypertension.* 2019;74 (4):1005-1013. doi: 10.1161/HYPERTENSIONAHA.118.12588.

81. Calderón-Pérez L, Gosalbes MJ, Yuste S, et al. Gut metagenomic and short chain fatty acids signature in hypertension: a cross-sectional study. *Sci Rep.* 2020;10 (1):6436. doi: 10.1038/s41598-020-63475-w.

82. Liu M, Nieuwdorp M, de Vos WM, Rampanelli E. Microbial Tryptophan Metabolism Tunes Host Immunity, Metabolism, and Extraintestinal Disorders. *Metabolites.* 2022;12 (9). doi: 10.3390/metabo12090834.

83. Soslau G. Cardiovascular serotonergic system: Evolution, receptors, transporter, and function. *J Exp Zool A Ecol Integr Physiol.* 2022;337 (2):115-127. doi: 10.1002/jez.2554.

84. Watts SW, Davis RP. 5-hydroxtryptamine receptors in systemic hypertension: an arterial focus. *Cardiovasc Ther.* 2011;29 (1):54-67. doi: 10.1111/j.1755-5922.2010.00173.x.

85. Watts SW, Morrison SF, Davis RP, Barman SM. Serotonin and blood pressure regulation. *Pharmacol Rev.* 2012;64 (2):359-388. doi: 10.1124/pr.111.004697.

86. Ala M, Eftekhar SP. The Footprint of Kynurenine Pathway in Cardiovascular Diseases. *Int J Tryptophan Res.* 2022;15:11786469221096643. doi: 10.1177/11786469221096643.

87. Fregly MJ, Sumners C, Cade JR. Effect of chronic dietary treatment with L-tryptophan on the maintenance of hypertension in spontaneously hypertensive rats. *Can J Physiol Pharmacol.* 1989;67 (6):656-662. doi: 10.1139/y89-105.

88. Lark LA, Witt PA, Becker KB, Studzinski WM, Weyhenmeyer JA. Effect of dietary tryptophan on the development of hypertension in the Dahl salt-sensitive rat. *Clin Exp Hypertens A.* 1990;12 (1):1-13. doi: 10.3109/10641969009074716.

89. Johansson ME, Jakobsson HE, Holmén-Larsson J, et al. Normalization of Host Intestinal Mucus Layers Requires Long-Term Microbial Colonization. *Cell Host Microbe.* 2015;18 (5):582-592. doi: 10.1016/j.chom.2015.10.007.

90. Durgan DJ, Ganesh BP, Cope JL, et al. Role of the Gut Microbiome in Obstructive Sleep Apnea-Induced Hypertension. *Hypertension.* 2016;67 (2):469-474. doi: 10.1161/HYPERTENSIONAHA.115.06672.

91. Knoop KA, Newberry RD. Goblet cells: multifaceted players in immunity at mucosal surfaces. *Mucosal Immunol.* 2018;11 (6):1551-1557. doi: 10.1038/s41385-018-0039-y.

92. Gustafsson JK, Johansson ME. The role of goblet cells and mucus in intestinal homeostasis. *NATURE REVIEWS GASTROENTEROLOGY & HEPATOLOGY.* 2022;19 (12):785-803. doi: 10.1038/s41575-022-00675-x.

93. Tyszko M, Lipinska-Gediga M, Lemanska-Perek A, Kobylinska K, Gozdzik W, Adamik B. Intestinal Fatty Acid Binding Protein (I-FABP) as a Prognostic Marker in Critically Ill COVID-19 Patients. *Pathogens.* 2022;11 (12). doi: 10.3390/pathogens11121526.

94. Gedgaudas R, Bajaj JS, Skieceviciene J, et al. Circulating microbiome in patients with portal hypertension. *Gut Microbes.* 2022;14 (1):2029674. doi: 10.1080/19490976.2022.2029674.

95. Li C, Xiao P, Lin D, et al. Risk Factors for Intestinal Barrier Impairment in Patients With Essential Hypertension. *Front Med (Lausanne).* 2020;7:543698. doi: 10.3389/fmed.2020.543698.

96. Xu X, Jin H, Li X, et al. Fecal Microbiota Transplantation Regulates Blood Pressure by Altering Gut Microbiota Composition and Intestinal Mucosal Barrier Function in Spontaneously Hypertensive Rats. *Probiotics Antimicrob Proteins.* 2024. doi: 10.1007/s12602-024-10344-x.

97. Yang T, Li H, Oliveira AC, et al. Transcriptomic Signature of Gut Microbiome-Contacting Cells in Colon of Spontaneously Hypertensive Rats. *Physiol Genomics.* 2020;52 (3):121-132.

98. Zundler S, Günther C, Kremer AE, Zaiss MM, Rothhammer V, Neurath MF. Gut immune cell trafficking: inter-organ communication and immune-mediated inflammation. *NATURE REVIEWS GASTROENTEROLOGY & HEPATOLOGY.* 2023;20 (1):50-64. doi: 10.1038/s41575-022-00663-1.

99. Li J, Richards EM, Handberg EM, Pepine CJ, Raizada MK. Distinct Gene Expression Profiles in Colonic Organoids from Normotensive and the Spontaneously Hypertensive Rats. *Cells.* 2021;10 (6). doi: 10.3390/cells10061523.

100. Franco M, Martinez F, Rodriguez-Iturbe B, et al. Angiotensin II, interstitial inflammation, and the pathogenesis of salt-sensitive hypertension. *Am J Physiol Renal Physiol.* 2006;291 (6):F1281-1287. doi: 10.1152/ajprenal.00221.2006.

101. Lu X, Rudemiller NP, Privratsky JR, et al. Classical Dendritic Cells Mediate Hypertension by Promoting Renal Oxidative Stress and Fluid Retention. *Hypertension.* 2020;75 (1):131-138. doi: 10.1161/HYPERTENSIONAHA.119.13667.

102. Madhur MS, Lob HE, McCann LA, et al. Interleukin 17 promotes angiotensin II-induced hypertension and vascular dysfunction. *Hypertension.* 2010;55 (2):500-507. doi: 10.1161/HYPERTENSIONAHA.109.145094.

103. Davis GK, Fehrenbach DJ, Madhur MS. Interleukin 17A: Key Player in the Pathogenesis of Hypertension and a Potential Therapeutic Target. *Curr Hypertens Rep.* 2021;23 (3):13. doi: 10.1007/s11906-021-01128-7.

104. Basile DP, Abais-Battad JM, Mattson DL. Contribution of Th17 cells to tissue injury in hypertension. *Curr Opin Nephrol Hypertens.* 2021;30 (2):151-158. doi: 10.1097/MNH.0000000000000680.

105. Saha P, Mell B, Golonka RM, et al. Selective IgA Deficiency in Spontaneously Hypertensive Rats With Gut Dysbiosis. *Hypertension.* 2022;79 (10):2239-2249. doi: 10.1161/HYPERTENSIONAHA.122.19307.

106. Aloysius A, DasGupta R, Dhawan J. The transcription factor Lef1 switches partners from β-catenin to Smad3 during muscle stem cell quiescence. *SCIENCE SIGNALING.* 2018;11 (540). doi: 10.1126/scisignal.aan3000.

107. Li J, Richards EM, Tummala R, Pepine CJ, Raizada MK, Yang T. Host-Microbiota Communication in Spontaneously Hypertensive Rats Generates Unique IgA-Coated Gut Microbes. *J Am Heart Assoc.* 2023;12 (4):e027918. doi: 10.1161/JAHA.122.027918.

108. Sharma RK, Yang T, Oliveira AC, et al. Microglial Cells Impact Gut Microbiota and Gut Pathology in Angiotensin II-Induced Hypertension. *Circ Res.* 2019;124 (5):727-736. doi: 10.1161/CIRCRESAHA.118.313882.

109. Seravalle G, Grassi G. Sympathetic nervous system and hypertension: New evidences. *Auton Neurosci.* 2022;238:102954. doi: 10.1016/j.autneu.2022.102954.

110. Shi P, Diez-Freire C, Jun JY, et al. Brain microglial cytokines in neurogenic hypertension. *Hypertension.* 2010;56 (2):297-303. doi: 10.1161/HYPERTENSIONAHA.110.150409.

111. Cheng L, Correia MLG. More Evidence Links Microglia and Neuroinflammation With Hypertension. *Am J Hypertens.* 2022;35 (9):787-789. doi: 10.1093/ajh/hpac081.

112. Mei X, Mell B, Manandhar I, et al. Repurposing a Drug Targeting Inflammatory Bowel Disease for Lowering Hypertension. *J Am Heart Assoc.* 2022;11 (24):e027893. doi: 10.1161/JAHA.122.027893.

113. Torres J, Bonovas S, Doherty G, et al. ECCO Guidelines on Therapeutics in Crohn's Disease: Medical Treatment. *J Crohns Colitis.* 2020;14 (1):4-22. doi: 10.1093/ecco-jcc/jjz180.

114. Donohoe DR, Garge N, Zhang X, et al. The microbiome and butyrate regulate energy metabolism and autophagy in the mammalian colon. *Cell Metab.* 2011;13 (5):517-526. doi: 10.1016/j.cmet.2011.02.018.

115. Kim S, Goel R, Kumar A, et al. Imbalance of gut microbiome and intestinal epithelial barrier dysfunction in patients with high blood pressure. *Clin Sci (Lond).* 2018. doi: 10.1042/CS20180087.

116. Li J, Richards EM, Handberg EM, et al. Influence of Butyrate on Impaired Gene Expression in Colon from Patients with High Blood Pressure. *Int J Mol Sci.* 2023;24 (3). doi: 10.3390/ijms24032650.

117. Dosoky NS, May-Zhang LS, Davies SS. Engineering the gut microbiota to treat chronic diseases. *Appl Microbiol Biotechnol.* 2020;104 (18):7657-7671. doi: 10.1007/s00253-020-10771-0.

118. Prasad R, Floyd JL, Dupont M, et al. Maintenance of Enteral ACE2 Prevents Diabetic Retinopathy in Type 1 Diabetes. *Circ Res.* 2023;132 (1):e1-e21. doi: 10.1161/CIRCRESAHA.122.322003.

119. Yang T, Maki KA, Marques FZ, et al. Hypertension and the Gut Microbiome: A Science Advisory From the American Heart Association. *Hypertension.* 2025. doi: 10.1161/HYP.0000000000000247.

120. Xiang T, Wang J, Li H. Current applications of intestinal organoids: a review. *Stem Cell Res Ther.* 2024;15 (1):155. doi: 10.1186/s13287-024-03768-3.

121. Taelman J, Diaz M, Guiu J. Human Intestinal Organoids: Promise and Challenge. *FRONTIERS IN CELL AND DEVELOPMENTAL BIOLOGY.* 2022;10. doi: 10.3389/fcell.2022.854740.

122. Abais-Battad J. Protective Effects of Female Gut Microbiota Transplantation on Hypertension and Renal Damage in Male Dahl Salt-Sensitive Rats. *PHYSIOLOGY.* 2023;38. doi: 10.1152/physiol.2023.38.S1.5733164.

123. Yang Y, Li J, Zhou Z, et al. Gut Microbiota Perturbation in Early Life Could Influence Pediatric Blood Pressure Regulation in a Sex-Dependent Manner in Juvenile Rats. *Nutrients.* 2023;15 (12). doi: 10.3390/nu15122661.

124. Virwani PD, Qian G, Hsu MSS, et al. Sex Differences in Association Between Gut Microbiome and Essential Hypertension Based on Ambulatory Blood Pressure Monitoring. *Hypertension.* 2023;80 (6):1331-1342. doi: 10.1161/HYPERTENSIONAHA.122.20752.

125. Chen L, He FJ, Dong Y, et al. Modest Sodium Reduction Increases Circulating Short-Chain Fatty Acids in Untreated Hypertensives: A Randomized, Double-Blind, Placebo-Controlled Trial. *Hypertension.* 2020;76 (1):73-79. doi: 10.1161/HYPERTENSIONAHA.120.14800.

126. Bardhan P, Mei X, Lai K, et al. Dysregulated Microbiota-indole Axis In Male, Not Female Ace2 Deficient Salt-sensitive Hypertension. *Hypertension.* 2023;80 (Suppl_1):A082-A082. doi: 10.1161/hyp.80.suppl_1.082.

127. Flood TR, Kuennen MR, Blacker SD, Myers SD, Walker EF, Lee BJ. The effect of sex, menstrual cycle phase and oral contraceptive use on intestinal permeability and ex-vivo monocyte TNFalpha release following treatment with lipopolysaccharide and hyperthermia. *Cytokine.* 2022;158:155991. doi: 10.1016/j.cyto.2022.155991.

128. Larauche M, Mahurkar-Joshi S, Biraud M, Ju T, Mayer EA, Chang L. Sex-dependent alterations of colonic epithelial permeability: relevance to irritable bowel syndrome. *Front Physiol.* 2025;16:1509935. doi: 10.3389/fphys.2025.1509935.

129. An Y, He Y, Ge N, Guo J, Yang F, Sun S. Organoids to Remodel SARS-CoV-2 Research: Updates, Limitations and Perspectives. *Aging Dis.* 2023;14 (5):1677-1699. doi: 10.14336/AD.2023.0209.

130. Barker N, van Es JH, Kuipers J, et al. Identification of stem cells in small intestine and colon by marker gene Lgr5. *NATURE.* 2007;449 (7165):1003-U1001. doi: 10.1038/nature06196.

131. Tian CM, Yang MF, Xu HM, et al. Stem cell-derived intestinal organoids: a novel modality for IBD. *Cell Death Discov.* 2023;9 (1):255. doi: 10.1038/s41420-023-01556-1.

132. Li J, Richards EM, Pepine CJ, et al. Reprograming of transcriptional profile of colonic organoids from patients with high blood pressure by minocycline. *Am Heart J Plus.* 2023;36. doi: 10.1016/j.ahjo.2023.100343.

**Figure Legend**

**Figure 1. Pathological changes associated with HTN.** Intestinal histopathological changes (top left) in HTN are characterized by increased gut fibrosis, muscular wall thickness, and villi dysplasia. These may lead to gut barrier disruption (top right) with decreased tight junction proteins and goblet cells, resulting in increased gut permeability, influx of gut microbiota-derived antigen, such as LPS, oxidative stress, and overactivated immune responses. The immune dysfunction (bottom left) includes both systemic and central immune activation with an increased release of pro-inflammatory cytokines. HTN-linked autonomic system imbalance (bottom right) includes both increased sympathetic activity and decreased parasympathetic activity. The red arrows represent the increased levels or activities in different pathologies, and the blue arrows represent the decreased levels or activities in different pathologies (Created in <https://BioRender.com>).

**Figure 2.** Pathological alterations in the gut lumen and lamina propria. Left panel, reduced release of acetylcholine by the vagal nerve results in less binding to α7nAChR expressed on multiple types of immune cells, thereafter more production of proinflammatory cytokines. Middle panel, dysfunctional goblet cells and reduced TJ increase gut permeability. This allows more LPS, derived from imbalanced gut microbiota, to cross the gut epithelium and activate innate immune cells, leading to release of proinflammatory cytokines, such as IL-1β. Subsequent activation of adaptive Th17 cells elevates levels of proinflammatory IL-17. Notably, IgA levels are deficient in SHR (not reported in other hypertensive rodents). Right panel, gut microbial imbalance significantly alters tryptophan metabolism, with increased utilization of tryptophan by gut microbiota for indole production, which reduces its conversion into kynurenine. In parallel, excessive salt intake contributes to both microbial imbalance and elevated blood pressure, partly through increased absorption via transporters such as NHE3.

α7nAChR, α7 nicotinic acetylcholine receptor; TJ, tight junction; LPS, lipopolysaccharide; IL-1β, interleukin-1β; IL-17, interleukin-17; IgA, immunoglobulin A; SHR, spontaneously hypertensive rat; DC, Dendritic; NHE3, sodium/hydrogen exchanger 3; Trp, tryptophan; TpH1, tryptophan hydroxylase 1; IDO1, indoleamine 2,3-dioxygenase-1 (Created in https://BioRender.com).

**Figure 3.** Proposed mechanisms for butyrate and minocycline regulating blood pressure via its impacts on the gut epithelium. Hypertension-associated gut pathology is partially attributed to imbalanced diet. Using the 3D intestinal organoids derived from hypertensive rats and human patients, studies show that butyrate and minocycline regulate the immune responses in the gut and increase TJ proteins, thereafter, contributing to lower blood pressure. Butyrate receptor GPRs 41, 43 and 109a may be involved. GPCRs, G-protein coupled receptors; TJ, tight protein.
